# IMPROVING GERIATRIC CARE OF INTERPROFESSIONAL PRACTICING PROVIDERS

**DOI:** 10.1093/geroni/igz038.1013

**Published:** 2019-11-08

**Authors:** Becky Powers, Jeanette Ross

**Affiliations:** 1 South Texas Veterans Health Care System Geriatric Research Education and Clinical Center, San Antonio, Texas, United States; 2 South Texas Veterans Health Care System, San Antonio, Texas, United States

## Abstract

Practicing providers often struggle with the care of older adults due to knowledge, skill, and attitude barriers. In an attempt to improve employee engagement in the care of older adults, the Geri-EMPOWER (Empowering Medical Providers and Older adults With strategies to Escape Readmission) program was initiated. In this program, case managers and visiting VA rural geriatric scholars participated in a two days of intensive educational sessions including lectures, shadowing inpatient teams and clinic providers, a dementia simulation learning exercise, and an Observed Structured Clinical Exam (OSCE) with standardized patient encounters. The 15 initial participating trainees came from a variety of medical backgrounds including physicians, nurse practitioners, nurses, social workers, and psychologists. A 10 item knowledge based pretest and posttest was constructed using the learning objectives of the course. Skills of attendees were directly observed during 4 OSCE stations. Attitudes towards older adults were measured before and after the intervention using the Caroline Opinions on Care of Older Adults (COCOA) scale. Geriatric and palliative care knowledge improved with average knowledge test scores improving from 63% to 86% before and after the course. Participants obtained all minimum competencies during their OSCE exam, and rated this session very highly in their course feedback. Attitudes towards older adults were also found to improve with an average COCOA score increase of 9 points before and after the educational sessions. This innovative course based in adult-learning theory demonstrates that employed interprofessional providers can quickly improve knowledge, skills, and attitudes towards older adults.

